# A deep dive into safety of breast implants and recreational diving: A systematic review

**DOI:** 10.1016/j.jpra.2025.08.020

**Published:** 2025-08-27

**Authors:** Elisa Maria Ragaini, Valeriano Vinci, Francesco Klinger, Flavio Bucci, Riccardo Di Giuli, Camilla Ferrari, Roberta Comunian, Marco Klinger, Stefano Vaccari

**Affiliations:** a Department of Medical Biotechnology and Translational Medicine BIOMETRA, Plastic Reconstructive and Aesthetic Plastic Surgery School, Università degli Studi Di Milano, Milan, Italy; b IRCCS Humanitas Research Hospital, via Manzoni 56 20089, Milan, Italy; c Department of Biomedical Sciences, Humanitas University, via Rita Levi Montalcini 4 20090, Pieve Emanuele, MI, Italy; d Department of Health Sciences, Ospedale San Paolo, University of Milan, Via Antonio di Rudinì, 8 20142, Milan, Italy

**Keywords:** Breast implants, Diving, Scuba diving, Hyperbaric pressure, Barometric changes, Implant integrity

## Abstract

**Objective:**

This review evaluates the performance and the safety of breast implants under conditions associated with recreational diving, focusing on their structural integrity, morphological changes, and long-term durability.

**Methods:**

A systematic review of the literature was performed. Included studies were those addressing effect of barometric pressure on breast implants. A comprehensive literature review from 1980 to 2024, including PubMed, Embase, Scopus, Medline, and Google Scholar databases, clinical experience, and case reports, is analyzed according to PRISMA guidelines.

**Results:**

Breast implants are advised as safe for recreational diving in standard depth limitations (<40 meters). Studies demonstrated no significant volume changes or ruptures under hyperbaric conditions. However, cohesive-gel implants exhibited some morphological alterations, particularly with repetitive dives, which could impact their long-term durability. Saline implants showed greater resistance to deformation compared to cohesive-gel and double-lumen implants.

**Discussion:**

The risk associated with recreational diving on breast implants is minimal, although factors such as frequency of diving, type of implant, and diving depth are important. Regular monitoring through imaging techniques and patient education can temper potential risks. Further research is needed to assess the long-term effects of diving on modern implants in real-world conditions.

**Conclusion:**

Breast implants are safe for recreational diving under typical conditions, but long-term durability may be affected by repetitive stress. Clinicians should provide personalized recommendations for divers while making sure to follow up regularly.

## Introduction

Recreational diving, a popular activity worldwide, poses unique physiological and mechanical challenges due to the varying pressures encountered during the descent and ascent. Women with breast implants  may be concerned about the safety, integrity, and durability of their implants due to the exposure to these pressure changes. Addressing these concerns is fundamental to ensure safe diving practices and to provide evidence-based guidance to patients and clinicians.

Breast implants are designed to withstand various physical stresses, including changes in pressure. Under increased external pressure, such as during diving, implants may experience compression, leading to temporary changes in shape. However, due to the cohesive nature of the silicone gel or the saline solution within the implants, they typically return to their original form once the pressure normalizes. This resilience ensures that implants maintain their integrity under typical pressure variations encountered during recreational diving.^1^^,^^2^

The relationship between implants and diving remains a poorly explored field, whereas their interaction with altitude has been extensively studied, particularly in the context of air travel. At high altitudes, with reduced atmospheric pressure, studies show that expansion of the implant can occur, however, this change is minimal and does not compromise the integrity of the implant. For instance, studies have demonstrated that silicone implants subjected to simulated high-altitude conditions (up to 35,000 feet) expand only slightly, contradicting the common belief of explosive rupture.^3^^,^^4^ Additionally, the U.S. food and drug administration (FDA) notes that physical stresses such as trauma or intense physical pressure can lead to complications with breast implants, but routine activities like flying are generally safe.^5^ As such, standard air travel does not pose a substantial risk to breast implant integrity.

Contrarily, the effects of hyperbaric conditions on breast implants have received far less attention in scientific research. The unique conditions of underwater pressure changes necessitate further investigation to provide comprehensive safety guidelines for divers with breast implants.

This review summarizes the current literature to evaluate the safety of breast implants in diving activities, focusing on structural integrity, potential risks, and long-term durability. By addressing the gaps in knowledge and providing a critical analysis of existing evidence, this review aims to inform clinical decision-making and reassure divers about the compatibility of implants with their underwater activities.

According to generally recognized international standards, we define “recreational diving” in this review as dives conducted for fun without decompression stops, usually with air or enriched air (nitrox), and up to 40 m deep.^6^ It's crucial to remember that, in reality, most recreational divers rarely go deeper than 18 to 30 m, with the great majority of dives taking place between 10 and 30 m.^7^

Furthermore, pressure rises nonlinearly with depth; at 10 m below the surface, it doubles (from 1 atm to 2 atm), but for every 10 m below that, it rises by about 1 atm. This affects tissue gas saturation, implant compression, and possible morphological changes, especially at shallower depths where the relative change is largest.

## Materials and methods

A systematic review of the literature was conducted following the guidelines outlined in the preferred reporting Items for systematic reviews and meta-analyses (PRISMA) protocol.^8^

The inclusion criteria for this review were based on the PICO framework, focusing on studies that specifically addressed the safety of breast implants in patients who practice diving. Studies were included if they: (1) Focused on breast implant performance under conditions of changing barometric pressure, including diving and altitude exposure; (2) Used experimental models, clinical trials, or observational studies to assess implant integrity, morphology, or volume; (3) Were published in peer-reviewed journals; (4) Were available in English.

Articles were excluded if they: (1) Did not provide original data (e.g., commentaries, editorials); (2) Focused on implants unrelated to breast augmentation (e.g., orthopedic or dental implants); (3) Lacked sufficient data or methodological transparency.

Articles were systematically reviewed from the PubMed, Embase, Medline, Google Scholar and Scopus databases, focusing on studies published from 1980 to 2024. The chosen search terms were “breast implants,” “diving,” “scuba diving,” “hyperbaric pressure,” “barometric changes,” and “implant integrity.” One author (EMR) conducted the initial assessment of the electronic search results and promptly excluded duplicate records. The remaining articles were then screened by three authors (EMR, SV, VV) based on the title, abstract, and publication type. A second round of screening, focusing on the content evaluation of the articles, was performed by the same authors. Any conflicts or discrepancies during this process were resolved by a fourth author.

**Figure 1:**
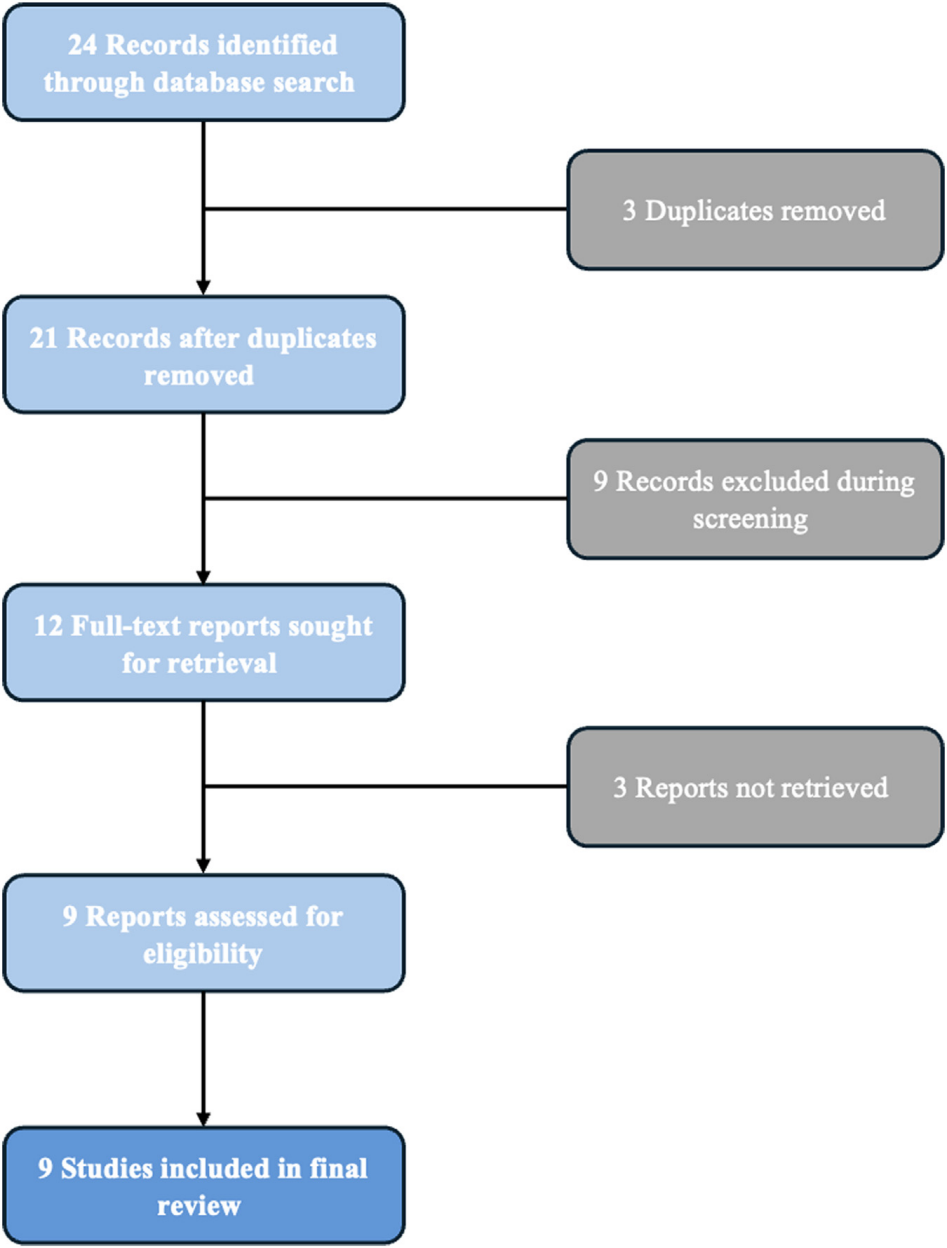
Flowchart of articles’ selection.

A total of 23 articles were identified through the database search, with two articles from PubMed, zero from Embase, one from Scopus, zero from Medline, and 20 from Google Scholar. After removing three duplicate records, 20 articles remained for screening. Of these, nine articles were excluded during the initial screening process for not meeting the inclusion criteria, such as lack of relevance to recreational diving or absence of original data. A total of 11 full-text reports were sought for retrieval, with three reports not retrieved due to accessibility issues. The three full-text articles could not be retrieved despite multiple attempts, including university library access, interlibrary loan requests, and direct author contact where possible. These studies were therefore excluded from final analysis due to lack of accessibility. This exclusion is acknowledged as a limitation of the present review. The remaining 8 reports were assessed for eligibility. After detailed review, all 8 studies met the inclusion criteria and were included in the final review. These studies were critically appraised for their design, methodology, and relevance to the research question Fig. 1.

## Literature review

### Breast implants and barometric pressure changes

Recreational diving exposes breast implants to continuous cycles of compression and decompression in response to changes in barometric pressure. These conditions can potentially influence implant integrity, morphology, and durability. Grippaudo et al.^1^^,^^9^ conducted a study to evaluate breast implants under simulated diving conditions in a hyperbaric chamber. They found that although implants retained their volume and integrity, some morphological changes were observed in cohesive-gel implants after multiple diving sessions raising concerns about long-term stress effects. In a complementary analysis by Verslegers et al.^10^ they examined the radiological response of implants to pressure variations using CT imaging, confirming that most implants maintain their integrity but may exhibit slight deformation under repetitive stress. The study by van Rappard^11^ adds further insight, showing that the pressure resistance of implants decreases slightly over time but remains sufficient to withstand barometric pressures encountered during recreational diving. They also demonstrated that older implants (implanted >5 years priors, often extending up to 10 or more years), may exhibit slightly reduced resistance to external pressures, though this decrease does not compromise safety under typical diving conditions.^11^ These results are consistent with previous studies, such as the one by Vann et al.^2^^,^^12^ which evaluated breast implants under extreme pressure conditions and found minimal volume changes, but highlighted the resistance of implant materials in recreational diving scenarios suggesting that diving may not pose a significant risk to implant integrity. Further studies on hyperbaric and hypobaric conditions have examined the wider implications of pressure changes, including altitude exposure after diving.^9^^,^^13^ Morphological changes, observed particularly under repetitive stress, underline the need for more robust materials and improved manufacturing techniques to enhance implant resistance in challenging environments.^14^^,^^15^

### Morphological alterations in implant materials

Changes in implant morphology under hyperbaric conditions were a recurring observation. For instance, Grippaudo et al.^1^ noted that cohesive-gel implants subjected to 40 simulated dives exhibited surface irregularities that persisted up to 12 months post-experiments, though functionality remained intact. Similarly, studies in the Diving and Hyperbaric Medicine Journal reported that double-lumen implants were more prone to irregularities, particularly around valve structures, suggesting they may be less suitable for divers.^14^ The study by van Rappard et al.^11^ further suggests that while older implants may undergo subtle structural changes over time, these do not significantly affect their performance in recreational diving scenarios. Studies on the effects of long-term aging of silicone implants suggest significant changes in mechanical properties, such as increased stiffness and reduced elongation, which could impact their ability to withstand repetitive stress from activities like diving.^16^

It is important to clarify how “deformation” was defined and assessed across the included studies. Deformation was defined by Grippaudo et al.^1^ as discernible surface irregularities and shape changes found during post-experimental visual and tactile examination of the implant surface following several simulated dives. Although there were no volume changes or ruptures, Verslegers et al. used CT imaging to identify small structural irregularities that they categorized as radiological deformations.^10^ Without mentioning any discernible alterations in shape or morphology, Van Rappard et al.^11^ concentrated on pressure resistance and recorded gradual declines in structural integrity.

However, none of the studies in this review assessed clinical outcomes like capsular contracture, explantation rates, patient-reported symptoms, or long-term follow-up data that linked morphological or radiological changes to patient experiences. Therefore, even though non-rupture structural changes—particularly in cohesive-gel implants—are frequently referred to as “deformation,” it is still unknown if these changes have any functional or clinical ramifications. A significant drawback of the existing literature is the absence of outcome data, which implies that observed changes should be interpreted with caution, especially when restricted to imaging results without patient-level data.

### Diving techniques and physiological considerations

Different diving techniques may influence the biomechanical and physiological stress imposed on breast implants. Apnea (breath-hold) diving and scuba (self-contained underwater breathing apparatus) diving differ in duration, depth, gas composition, and decompression patterns. While apnea dives are typically shallow (<20 m) and of short duration, scuba diving may involve longer exposures and deeper depths (commonly up to 30–40 m). These differences affect the rate and extent of gas absorption and decompression, which are critical when evaluating potential risks for implant deformation or surrounding tissue changes.^17^^,^^18^

Depending on exposure and depth, scuba divers may use gas mixtures like trimix (He–N₂–O₂), enriched air nitrox (EANx), or air (21% O₂). Henry's Law states that these gases dissolve in tissues, and supersaturation during ascent may cause microbubbles to form in tissues and encapsulated spaces. Dissolved gases are absent from implants, but they may have an impact on the surrounding periprosthetic tissues. On the other hand, because apnea diving is brief, it causes less gas absorption even though it is linked to quick pressure changes. On the other hand, “serial diving,” or rapid succession of deep apnea dives, may raise the risk of decompression stress.^15^^,^^19^

These differences highlight the importance of considering diving modality when assessing implant safety.

Unfortunately, current studies do not differentiate between these techniques, and future research should aim to stratify diving profiles more precisely.

### Risk factors in implant safety

Key risk factors affecting implant safety during diving include:

- Repetitive stress: studies by Colombo et al. and Vann et al.^2^^,^^9^ show that the likelihood of morphological changes is greater in patients undergoing repeated diving, especially those who have implants with older materials or designs.
- Depth and duration of dives: recreational dives of less than 40 m in depth seem to have no significant risks. Yet deeper or longer dives may promote increased stress on implants.^10^^,^^14^
- Implant type: cohesive-gel implants are more durable than saline ones, but also show a higher tendency towards shape distortions.^1^^,^^15^

### Implications for divers with breast implants

Though the evidence is still limited, it suggests that recreational diving with breast implants is safe. Nonetheless, divers should focus on regular follow-ups, especially for individuals with older implants and on avoiding extreme diving conditions to minimize stress on implants.^1^^,^^9^^,^^10^

### Physiological effects of repeated pressure cycles and impact on periprosthetic tissues

In addition to the direct mechanical effects on the implant, the periprosthetic environment may be impacted by systemic and local pathophysiological changes brought on by repeated cycles of compression and decompression during diving. Under hyperbaric conditions, the human body undergoes notable changes in oxidative stress, vascular perfusion, and gas solubility, all of which can affect tissue homeostasis. The fibrous capsule that naturally develops around every breast implant is of special interest. Its structure, vascularization, and inflammatory profile may change as a result of repeated pressure exposure, according to studies. A recent study by Janszen et al.^16^ showed that in a rat model, exposure to hyperbaric oxygen therapy (HBOT) dramatically decreased capsular thickness, indicating that hyperbaric environments may have a modulatory effect on fibrotic processes. Although recreational diving and HBOT are not the same, they both use hyperbaric principles, which makes it worthwhile to investigate how diving might affect the tissue responses surrounding implants over the long term.

Furthermore, systemic effects that may indirectly affect wound healing, capsule formation, and tissue resilience include endothelial activation, nitric oxide modulation, and reactive oxygen species (ROS) production during and after diving.^19^^,^^20^ These physiological pathways deserve further investigation, particularly in frequent divers with implants.

The findings from this review suggest that breast implants are generally safe for recreational diving, with minimal risks of rupture or significant volume changes under typical diving conditions. Overall, the studies analyzed point to unique considerations and constraints, particularly with respect to morphological alterations and the chronic stability of specific categories of implants.

The study by van Rappard et al.^11^ supported the notion that breast implants retain their structural integrity and pressure resistance over time, even under conditions associated with recreational diving. While slight reductions in pressure resistance were observed with older implants, these changes remained within safe limits for typical diving scenarios. Some studies, including Grippaudo et al.^1^ have noted morphological alterations in cohesive-gel implants subjected to repetitive diving conditions. Although the described changes did not compromise implant integrity, their persistence in the following year underlines that repetitive and cumulative stresses of diving can affect the implant structure in the long term. Additional investigations with radiological analyses confirmed that repeated barometric pressure changes can lead to minor irregularities in implant morphology.^9^ These findings imply that, even though short-term diving has negligible risks, long-term exposure to repetitive diving needs to be monitored, particularly in active divers.

The type of implant material is also an essential factor in determining its resistance to hyperbaric conditions. Compared to saline implants, cohesive-gel implants displayed greater morphological deformation.^1^^,^^15^

The behavior of double-lumen implants under simulated hyperbaric conditions was examined in one study by Szkurlat et al.^14^ which identified possible weaknesses in particular structural regions. The authors specifically detailed findings from post-dive assessments and laboratory simulations that revealed surface irregularities and localized deformities surrounding the valve structures of double-lumen devices. Several hyperbaric pressure cycles were applied to different implant types in a controlled chamber as part of the experimental setup, and surface examinations were then conducted. These alterations raised questions regarding the long-term structural resilience even though they were not linked to rupture or volume loss. It is crucial to emphasize that this conclusion is predicated on a small amount of in vitro testing and lacks clinical correlation and long-term monitoring.

While the risk of rupture or significant deformation is low, clinicians should counsel patients with breast implants who engage in frequent diving. Szkurlat emphasized the importance of regular follow-ups to monitor implant integrity and identify early signs of morphological changes or wear.^14^ Moreover, divers should be advised to avoid extreme depths exceeding 40 m, as deeper dives impose additional pressures that could exacerbate stress on implants.^1^^,^^14^

While the current evidence provides valuable insights, significant gaps remain.

From a physical standpoint, it's crucial to remember that the main components of breast implants, silicone gel and saline, are both essentially made of incompressible fluids. Unlike air-filled structures, this feature implies that implants should, in theory, retain their volume and shape under pressure.^5^ Moreover, according to Henry’s Law, the amount of a gas that dissolves in a liquid is proportional to the partial pressure of that gas above the liquid. During descent in diving, increased ambient pressure elevates gas solubility in tissues, while ascent leads to supersaturation and potential bubble formation. Although breast implants do not contain gas, per se, localized formation of microbubbles in adjacent tissues or encapsulated spaces has been observed in high-altitude simulations and may be relevant to diving conditions as well.^15^^,^^19^

Diving is the opposite of high-altitude exposure, where low external pressure may cause implant expansion; elevated external pressure may cause temporary compression. Nonetheless, it is still worthwhile to research the buildup and release of dissolved gases during decompression, especially in light of implant-tissue interfaces and capsule behavior.^3^^,^^4^

The majority of studies primarily examined simulated diving environments, and may not accurately reflect the complexities of actual underwater diving situations.^1^^,^^9^ Moreover, there is a lack of recent studies addressing this topic, as most of the ones present in literature date back to over a decade ago. Future research is needed, focusing on long-term studies on active divers to better understand the cumulative effects of repeated diving Table 1.

**Table 1:** Summary of the included articles.

Reference	Study type	Depth/Pressure simulated	Aim	Outcomes
Grippaudo et al.^1^	Experimental	Simulated dives (up to 40m, multiple cycles)	To evaluate the structural integrity and morphology of breast implants under simulated diving conditions.	No rupture or volume changes; morphological alterations observed in cohesive-gel implants.
Verslegers et al.^10^	Experimental	∼2–4 atm (CT pressure simulation)	To assess alterations in breast implants exposed to barometric pressure changes using CT imaging.	CT imaging detected minor structural changes in some implants but no ruptures.
Van Rappard et al.^11^	Experimental	3–6 atm; correlation with implantation time	To evaluate the pressure resistance of breast implants as a function of implantation time.	Pressure resistance slightly decreases with implantation time but remains within safe limits for recreational diving. Older implants showed minor reductions in structural integrity, but no critical failures under tested conditions.
Vann et al. ^2^	Experimental	Hyperbaric chamber simulation (∼4 atm)	To study the effects of diving and altitude exposure on mammary implants.	Minimal volume changes; implants maintained integrity under recreational diving conditions.
Pearson et al. ^12^	Clinical Study	Variable conditions (not precisely defined)	To analyze silicone breast implants under variable conditions.	Reported the durability and resistance of implants under stress conditions.
Grippaudo et al.^9^	Experimental – preliminary data	Simulated dives (∼3–4 atm)	To present findings on breast implants under simulated diving conditions.	Preliminary results suggested safety for recreational diving; morphological changes noted.
Hart^13^	Review	Not applicable	To address complications of breast implants in clinical scenarios.	Highlighted management of implant complications, emphasizing clinical strategies.
Szkurlat^14^	Review	Not applicable	To discuss diving and hyperbaric conditions relevant to breast implants.	Provided a general overview of hyperbaric conditions affecting implant behavior.
Edge ^15^	Review	General diving risks; not quantified	To explore medical conditions, including breast implants, that affect diving risks.	Identified risk factors for divers with implants; emphasized safety measures.

## Conclusion

The literature suggests that breast implants per se are generally safe for recreational diving and risks are negligible in normal conditions. Yet, morphological alterations noted in specific implant types, especially under repetitive stress, emphasize the need for  frequent follow-up examinations as well as patient education. Further research in real-world diving scenarios is critical in addressing remaining uncertainties and optimizing care for divers with breast implants.

## Declaration of competing interest

The authors declare that they have no known competing financial interests or personal relationships that could have appeared to influence the work reported in this paper.
